# Nervous Necrosis Virus Replicates Following the Embryo Development and Dual Infection with Iridovirus at Juvenile Stage in Grouper

**DOI:** 10.1371/journal.pone.0036183

**Published:** 2012-04-26

**Authors:** Hsiao-Che Kuo, Ting-Yu Wang, Hao-Hsuan Hsu, Peng-Peng Chen, Szu-Hsien Lee, Young-Mao Chen, Tieh-Jung Tsai, Chien-Kai Wang, Hsiao-Tung Ku, Gwo-Bin Lee, Tzong-Yueh Chen

**Affiliations:** 1 Laboratory of Molecular Genetics, Institute of Biotechnology, National Cheng Kung University, Tainan, Taiwan; 2 Research Center of Ocean Environment and Technology, National Cheng Kung University, Tainan, Taiwan; 3 Agriculture Biotechnology Research Center, National Cheng Kung University, Tainan, Taiwan; 4 Institute of Nanotechnology and Microsystems Engineering, National Cheng Kung University, Tainan, Taiwan; 5 Department of Engineering Science, National Cheng Kung University, Tainan, Taiwan; 6 Division of Environmental Health and Occupational Medicine, National Health Research Institutes, Zhunan, Miaoli, Taiwan; 7 Research Division I, Taiwan Institute of Economic Research, Taipei, Taiwan; 8 Office for Energy Strategy Development, National Science Council, Taipei, Taiwan; 9 Department of Power Mechanical Engineering, National Tsing Hua University, Hsinchu, Taiwan; Institut Pasteur, France

## Abstract

Infection of virus (such as nodavirus and iridovirus) and bacteria (such as *Vibrio anguillarum*) in farmed grouper has been widely reported and caused large economic losses to Taiwanese fish aquaculture industry since 1979. The multiplex assay was used to detect dual viral infection and showed that only nervous necrosis virus (NNV) can be detected till the end of experiments (100% mortality) once it appeared. In addition, iridovirus can be detected in a certain period of rearing. The results of real-time PCR and *in situ* PCR indicated that NNV, in fact, was not on the surface of the eggs but present in the embryo, which can continue to replicate during the embryo development. The virus may be vertically transmitted by packing into eggs during egg development (formation) or delivering into eggs by sperm during fertilization. The ozone treatment of eggs may fail to remove the virus, so a new strategy to prevent NNV is needed.

## Introduction

There are two common seen viruses in farmed groupers. Nervous necrosis virus (NNV), a two-single-stranded RNA piscine nodavirus, can cause damage to the central nervous system [1], [2] and results in high mortality rates (80–100%) of hatchery-reared larvae and juveniles [3]–[6]. Iridoviruses, a virus with double-stranded DNA [7], [8], can cause serious diseases in poikilothermic vertebrates, fish, amphibians, or reptiles [9] and have had a significant negative impact on modern aquaculture and wildlife conservation [10]. In the laboratory, intraperitoneal challenge of healthy juvenile grouper with grouper iridovirus resulted in cumulative mortality of 100% within 11 days [11]. Since 1995, this virus has almost caused epizootics in grouper fish farms of southern Taiwan, where it has caused up to 60% mortality [11]. In addition, farmed groupers are susceptible to infection by *Vibrio anguillarum*, a bacterial pathogen found in marine and freshwater fish species which can cause a terminal hemorrhagic septicemia known as vibriosis leading to high mortality rates [12], [13].

It has been known that piscine nodavirus can be transmitted vertically from the broodfish via the eggs [1], [14], [15]. The ozone and other chemicals have been applied to treat the eggs in order to break virus particles on the surface of the eggs and produce virus-free eggs [16]. However, juveniles from ozone-treated eggs still have high mortality due to virus infection. In the case of Atlantic halibut, *Hippoglossus hippoglossus*, disinfection by ozonation of sea water still has the cumulative mortality rate of 100% to larvae in 44 days [17]. The high mortality rate makes the NNV transmission pathway remain a mystery.

To reveal this mystery, our efforts have focused on the development of microfluidics-based multiplex RT-PCR for detection of pathogens in farmed fish [18], specifically nodavirus, iridovirus and *V. anguillarum* which mainly cause diseases to grouper fish. Initially, to understand the infection status of different pathogens and possible interactions in grouper fish farm we had applied the multiplex RT-PCR system. The present study also incorporates detection of expression of the Mx gene. This sequence encodes a cytoplasmic protein with activity against a number of viruses [19], [20]. Interferon can induce the Mx gene expression which has also been used as a molecular marker for type I interferon production [21], [22]. Similarly, grouper Mx gene expression is induced by piscine viruses (e.g., nodavirus and iridovirus) infection but not by bacterial infection; thus, Mx gene expression can be used to monitor viral infection in grouper [23].

From the information regarding these infectious pathogens within fish farm, we were aiming to identify the vertical transmission pathway of NNV.

## Results

### Evaluation of multiplexing assay

**Figure 1:**
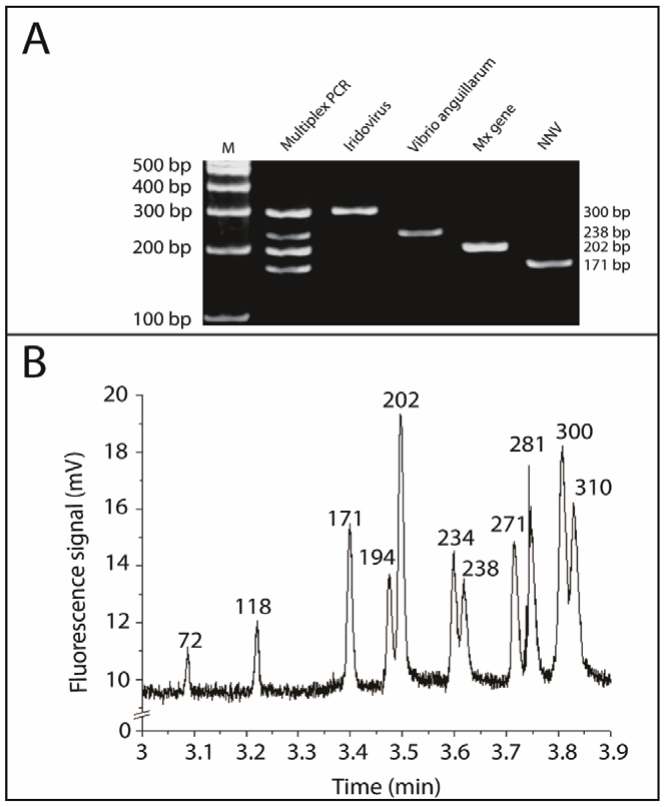
Optimization of the multiplex RT-PCR system. The microfluidic RT-PCR system is comprised of a microfluidic control module and a micro temperature control module [18]. The current design of microfluidic system is capable for rapid detection of four target genes (two viruses, one bacteria and one host immune response gene). The cDNA of samples can be uniformly distributed into the subsequent four PCR reaction chambers to detect four genes simultaneously. **A**. 100 ng of cDNA was amplified with each set of primers separately and with the mixture of the primers. Lane 2, Iridovirus; lane 3, *Vibrio anguillarum*; lane 4, Mx; lane 5, NNV (nodavirus); lane 1, multiprimer reactions simultaneously in a single tube. **B**. The electropherograms of the RT-PCR products from the single-tube reaction. The sizes of the DNA marker are 310 bp, 281 bp, 271 bp, 234 bp, 194 bp, 118 bp and 72 bp.

The microfluidic chip device [18] can distinguish the amplified pathogen signals from the DNA marker (Figure S2). An ethidium bromide-stained gel of the four fragments, amplified by the RT-PCR reactions are shown in Fig. 1A. 300-bp, 238-bp, 202-bp and 171-bp fragments were observed as expected, corresponding to products amplified from sequences encoding major capsid protein of iridovirus, flagellin A of *V. anguillarum*, grouper Mx, and RdRp and protein B2 of nodavirus. When all four templates and primer pairs were pooled and subjected to RT-PCR, four bands of the expected sizes were present and clearly distinguished (Fig. 1A, lane 1). The same results were obtained by CE on separation of four signals from pathogens and Mx gene (Fig. 1B).

### Evaluation of assay on *ex vivo* samples

The results showed that the amplified PCR products (Figure S3) were expected regarding to challenging experiments. NNV was detected in group 1 fish (lane 1, Fig. S3), *V. anguillarum* was detected in group 2 fish (lane 2, Fig. S3), iridovirus was detected in group 3 fish (lane 3, Fig. S3), NNV and *V. anguillarum* were detected in group 4 fish (lane 4, Fig. S3), NNV and iridovirus were detected in group 5 fish (lane 5, Fig. S3), iridovirus and *V. anguillarum* were detected in group 6 (lane 6, Fig. S3), NNV, iridovirus and *V. anguillarum* were detected in group 7 fish (lane 7, Fig. S3).

### Monitoring of pathogens contamination in fish farm

**Figure 2:**
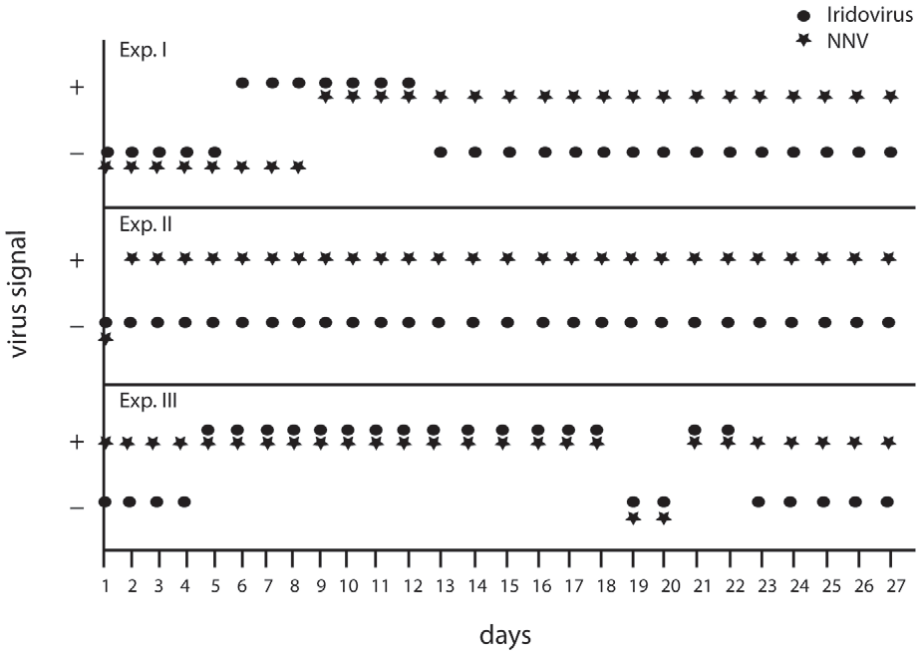
The RT-PCR on microfluidic chip to detect irodovirus and nordavirus (NNV) during juvenile rearing. Three experiments (Exp. I, Exp. II and Exp. III) have been proceed, sampled and monitored continuously for 27 days for each experiments.

The multiplex RT-PCR method was applied to investigate the infection status of individual fish rearing tank for three times, and each time for sampling continuously in 27 days (three repeats in Table 1). The RT-PCR assay did not detect *V. anguillarum* in any of these grouper fish samples but Mx gene expression was detected in most of the cases (except Exp. II-23 and Exp. III-19 were shown as negative). In Exp. I, iridovirus was first detected on the 6^th^ day while NNV was detected on the 9^th^ day. The NNV signal was detected continuously for the rest of the experiment but the signal of iridovirus only appeared for one week (day 6 to day 12) (Figure 2). Different story was observed in Exp. II, no iridovirus was detected but NNV signal came out from the second day of the experiment (Figure 2). In Exp. III, NNV signal appeared from the first day of the experiment. In this experiment, iridovirus was detected from day 5 to day 22 of the experiment. However, in Exp. III, both iridovirus and NNV signals were not detected on the 19^th^ and the 20^th^ day of the experiment (Figure 2). This may be due to sampling and false negative results.

**Table 1:** Results of mutliplex RT-PCR assay for individual aquaculture farms.

	Presence of PCR Product				
Sample‡	Nodavirus	Iridovirus	*V. anguillarum*	Mx gene	Date (day/mon/yr)
Exp. I					
Exp.I-1	**−**	**−**	**−**	**+**	16/12/2008
Exp.I-2	**−**	**−**	**−**	**+**	17/12/2008
Exp.I-3	**−**	**−**	**−**	**+**	18/12/2008
Exp.I-4	**−**	**−**	**−**	**+**	19/12/2008
Exp.I-5	**−**	**−**	**−**	**+**	20/12/2008
Exp.I-6	−	+	**−**	+	21/12/2008
Exp.I-7	−	+	**−**	+	22/12/2008
Exp.I-8	**−**	+	**−**	+	23/12/2008
Exp.I-9	**+**	**+**	**−**	**+**	24/12/2008
Exp.I-10	**+**	**+**	**−**	**+**	25/12/2008
Exp.I-11	**+**	**+**	**−**	**+**	26/12/2008
Exp.I-12	**+**	**+**	**−**	**+**	27/12/2008
Exp.I-13	**+**	**−**	**−**	**+**	28/12/2008
Exp.I-14	**+**	**−**	**−**	**+**	29/12/2008
Exp.I-15	**+**	**−**	**−**	**+**	30/12/2008
Exp.I-16	**+**	**−**	**−**	**+**	31/12/2008
Exp.I-17	**+**	**−**	**−**	**+**	01/01/2009
Exp.I-18	**+**	**−**	**−**	**+**	02/01/2009
Exp.I-19	**+**	**−**	**−**	**+**	03/01/2009
Exp.I-20	**+**	**−**	**−**	**+**	04/01/2009
Exp.I-21	**+**	**−**	**−**	**+**	05/01/2009
Exp.I-22	**+**	**−**	**−**	**+**	06/01/2009
Exp.I-23	**+**	**−**	**−**	**+**	07/01/2009
Exp.I-24	**+**	**−**	**−**	**+**	08/01/2009
Exp.I-25	**+**	**−**	**−**	**+**	09/01/2009
Exp.I-26	**+**	**−**	**−**	**+**	10/01/2009
Exp.I-27	**+**	**−**	**−**	**+**	11/01/2009
Exp. II					
Exp.II-1	**−**	**−**	**−**	**+**	16/02/2009
Exp.II-2	**+**	**−**	**−**	**+**	17/02/2009
Exp.II-3	**+**	**−**	**−**	**+**	18/02/2009
Exp.II-4	**+**	**−**	**−**	**+**	19/02/2009
Exp.II-5	**+**	**−**	**−**	**+**	20/02/2009
Exp.II-6	**+**	**−**	**−**	**+**	21/02/2009
Exp.II-7	**+**	**−**	**−**	**+**	22/02/2009
Exp.II-8	**+**	**−**	**−**	**+**	23/02/2009
Exp.II-9	**+**	**−**	**−**	**+**	24/02/2009
Exp.II-10	**+**	**−**	**−**	**+**	25/02/2009
Exp.II-11	**+**	**−**	**−**	**+**	26/02/2009
Exp.II-12	**+**	**−**	**−**	**+**	27/02/2009
Exp.II-13	**+**	**−**	**−**	**+**	28/02/2009
Exp.II-14	**+**	**−**	**−**	**+**	01/03/2009
Exp.II-15	**+**	**−**	**−**	**+**	02/03/2009
Exp.II-16	**+**	**−**	**−**	**+**	03/03/2009
Exp.II-17	**+**	**−**	**−**	**+**	04/03/2009
Exp.II-18	**+**	**−**	**−**	**+**	05/03/2009
Exp.II-19	**+**	**−**	**−**	**+**	06/03/2009
Exp.II-20	**+**	**−**	**−**	**+**	07/03/2009
Exp.II-21	+	**−**	**−**	**+**	08/03/2009
Exp.II-22	+	**−**	−	**+**	09/03/2009
Exp.II-23	+	**−**	**−**	**−**	10/03/2009
Exp.II-24	**+**	**−**	**−**	**+**	11/03/2009
Exp.II-25	**+**	**−**	**−**	**+**	12/03/2009
Exp.II-26	**+**	**−**	−	**+**	13/03/2009
Exp.II-27	**+**	−	**−**	**+**	14/03/2009
Exp. III					
Exp.III-1	**+**	**−**	**−**	**+**	18/04/2009
Exp.III-2	**+**	**−**	**−**	**+**	19/04/2009
Exp.III-3	**+**	**−**	**−**	**+**	20/04/2009
Exp.III-4	**+**	**−**	**−**	**+**	21/04/2009
Exp.III-5	**+**	+	**−**	**+**	22/04/2009
Exp.III-6	**+**	**+**	**−**	**+**	23/04/2009
Exp.III-7	**+**	+	**−**	**+**	24/04/2009
Exp.III-8	**+**	**+**	**−**	**+**	25/04/2009
Exp.III-9	**+**	+	**−**	**+**	26/04/2009
Exp.III-10	**+**	+	**−**	**+**	27/04/2009
Exp.III-11	**+**	+	**−**	**+**	28/04/2009
Exp.III-12	**+**	+	**−**	**+**	29/04/2009
Exp.III-13	+	+	**−**	**+**	30/04/2009
Exp.III-14	+	**+**	**−**	**+**	01/05/2009
Exp.III-15	+	+	**−**	**+**	02/05/2009
Exp.III-16	**+**	+	**−**	**+**	03/05/2009
Exp.III-17	+	+	**−**	**+**	04/05/2009
Exp.III-18	+	+	**−**	**+**	05/05/2009
Exp.III-19	**−**	**−**	**−**	**−**	06/05/2009
Exp.III-20	**−**	**−**	**−**	**+**	07/05/2009
Exp.III-21	+	+	−	**+**	08/05/2009
Exp.III-22	+	+	−	**+**	09/05/2009
Exp.III-23	**+**	**−**	**−**	**+**	10/05/2009
Exp.III-24	**+**	**−**	**−**	**+**	11/05/2009
Exp.III-25	**+**	**−**	**−**	**+**	12/05/2009
Exp.III-26	**+**	**−**	**−**	**+**	13/05/2009
Exp.III-27	**+**	**−**	**−**	**+**	14/05/2009

‡ All of the surveyed sites were indoor grouper aquaculture farms with maintained seawater at controlled temperature (29°C) and pH (7.37 to 7.49). The location and species were Linyuan(LY) and *E. coioides*, respectively.

Total number of fish sampled: Six fish were collected and pooled for each site.

### NNV continued to replicate and localized in the embryo

**Figure 3:**
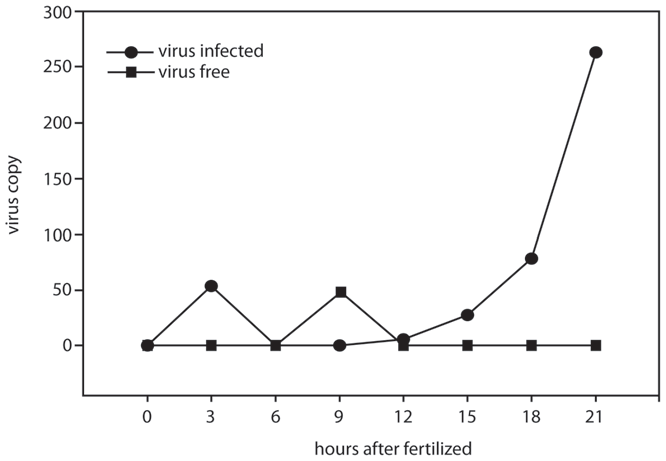
Real-time PCR of NNV-free and NNV-infected grouper eggs. The amount of virus was increased following the development of embryo.

The amount of virus was increased during the development of the embryo (Figure 3) and even after they hatch (data not shown).

**Figure 4:**
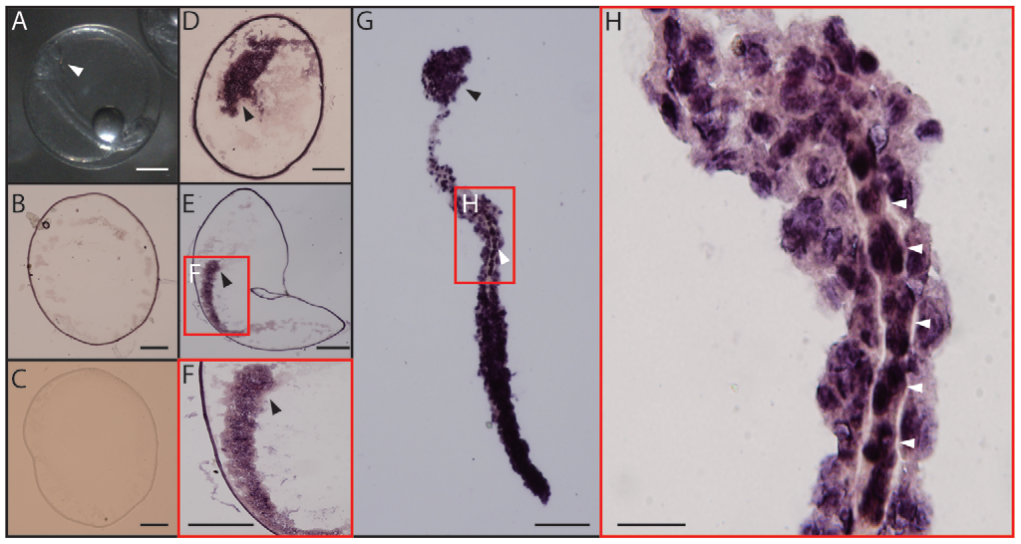
*In situ* RT-PCR of grouper eggs (15 h after spawning). **A**, grouper egg. The embryo has been developed. White arrow indicated the head of the embryo. **B**, section of non-fertilized grouper egg (no embryo). **C**, section of virus-free grouper egg (negative control). **D–F**, section of virus-containing grouper egg (positive control). The dark precipitation indicated the signal of NNV. Black arrow heads indicated the heads of the embryos. Figure 4F was the magnification image from the red box in the figure 4E. **G–H**, section of virus-containing grouper embryo. The dark precipitation indicated the signal of NNV (Black arrow head; also indicated the head of the embryo) Figure 4H was the magnification image from the red box in the figure 4G. The spinal cord can be visualized in **H** (white arrow heads) the middle line of the embryo) and showed the signal of NNV. Bar = 0.2 µm (**A–G**); bar = 0.1 µm (**H**).

The results also showed that, NNV is localized inside the embryo but not on the surface of the egg (Figure 4D–4H). Unfertilized (Figure 4B) and NNV-free eggs (Figure 4C) showed no signal of NNV (Figure 4B–4H).

## Discussion

The major recently reported pathogens, e.g., iridovirus, NNV and *V. anguillarum*, to grouper fish were chosen to be investigated by microfluidic chip in this study. The assay also incorporates a host immune response marker, Mx which encodes an interferon-induced member of the dynamin superfamily of large GTPases [23], [24]. The gene can be used as an indicator to monitor fish to general viral exposure, such as latent or unknown viral pathogens. Vibriosis, caused by *V. anguillarum*, was an important infectious disease in cultured marine fish in Taiwan in the late 1970s and early 1980s [25]. In the present survey however, no *V. anguillarum* infection was detected, indicated the improvements of grouper aquaculture over the past 30 years. Circulating and regular exchange of fresh sea water can maintain the bacteria below disease-causing concentration in culturing tank. In addition, the improvement of fish farm management (record and regular check and identify for pathogens) is also a key factor to prevent the disease caused by bacteria.

Although vibriosis may have almost been terminated in farmed groupers, other diseases caused by virus are still causing trouble to the grouper aquaculture. To solve this problem, advanced techniques have been introduced to modern aquaculture for detection and monitoring the potential viral pathogens. Here, we introduced the microfluidic chip system to grouper aquaculture. The microfluidic chip system is able to do multiplex assay. Instead of previous target RNA2 of nodavirus [3], the overlapping sequence between RNA1 and RNA3 was chosen for this study. The advantages of using this target in the current work were, (1) the size of the intended PCR product had to be distinct from each other, (2) the sequence of the primer pairs had to be compatible with other primer pairs in the “one-pot” PCR reaction,(3) the PCR conditions had to be the same for all the primer sets and the most important of all was that RNA1 and RNA3 are both expressed in the early stage of infection [26].

The multiplex assay also detected dual viral infection (Figure 2, Exp. I and Exp. III). In the case of mosquito cells, it can accommodate at least 3 viruses simultaneously, which provides an opportunity for genetic exchange between diverse viruses [27]. Different strains of virus from different hosts may also be due to genetic exchange. Interestingly, in our observation, only NNV can be detected till the end (100% mortality) of experiments once it appeared. For the other detection, iridovirus was not detected in Exp. II but could be detected in certain period of time (6^th^–12^th^ day in Exp. I and 5^th^–22^nd^ in Exp. III). This phenomenon needs to be investigated in more detail to clarify whether NNV can influence the replication of iridovirus or not.

We have demonstrated that the problem caused by NNV in grouper aquaculture is much more serious than by iridovirus. It was reported that vertical transmission is the only infection pathway for salmon anemia virus (ISAV) [28], [29]. In the case of NNV in grouper fish farming, the difficulty to remove the virus from fish farm may be due to the vertical transmission pathway involved in spread of the virus. It has been suggested that the treatment of grouper eggs with ozone or other chemicals can remove the virus from the eggs [16]. However, juveniles from ozone-treated eggs can still be infected by the virus, and the cumulative mortality rate is even close to 100% [17](our own observation, data not shown). In fact, NNV is not localized on the surface of the eggs (Figure 4) but it is present inside the embryo and may continue to replicate during the embryo development (Figures 3 and 4).

The virus may have been packed into the eggs since egg development (formation) or it has been delivered into the eggs by the sperms during fertilization. The research on hepatitis B virus has shown that the viral DNA can pass through the zona and oolemma and enter into oocytes by sperm [30], [31]. Furthermore, it was also reported that the virus can be absorbed into the fish sperm and vertically transmitted [32]. Or alternatively, the NNV genome might integrate into host genome. There were evidences indicating that some virus species, such as adenoviral vectors, could integrate into host genome and pass to embryo through vertical transmission pathway [33]. Likewise, HBV and HIV-1 also can integrate into host embryonic genome [30], [34]. Grouper genome containing NNV genome also can explain why most of the broodstocks and wild marine fish are positive for betanodavirus [35]. The results from Mx gene expression (Exp. I, 1^st^–5^th^ day) showed in some cases that the gene was highly expressed without viral (neither NNV nor iridovirus) infection. These results may due to the physical status of when the viral infection gets started or close to the end of sampling individuals. In mammalian, when treated with interferon (mimic the virus infection), Mx was detectable in 3 h, and its expression had continuously increased for another 5 h and decreased thereafter [36]. Moreover, Mx could not be detected before 3 h and after 20 h [36].

In conclusion, NNV can vertically transmit from parental grouper fish to the progenies at the embryo stage. The virus could be brought into the egg by sperm during fertilization. Or one possibility was that the virus genome may have already integrated into some of the host fish genome. The ozone treatment of eggs may fail to remove the virus as there is no sign of virus on the surface of the egg. The grouper fish genome (approximately 1 Giga-bases) sequencing has been initiated in our lab which will be completed recently. The genome information should lead to a better understanding of the interactions between the pathogens and host.

## Materials and Methods

### Grouper fish sampling and challenge

*Epinephelus coioides* (orange-spotted grouper) were collected from a grouper fish farm in Linyuan, Taiwan from 2008 to 2009 (Table 1). Three individual experiments were performed in 3–4 weeks and continuously sampled for 27 days. The juveniles used were 25–30 days post hatching. For each sample, six live juveniles were randomly selected (whole fish which were then killed and pooled before submitted to experiment) and immersed in TRIzol Reagent™ (Molecular Research Center, Inc. Ohio, USA) solution directly. Twelve asymptomatic groupers ranging in size from 0.5–3 inches were obtained from grouper aquaculture farm and maintained in a 1×0.5×0.5 m^3^ fish tank with constant aeration and a water temperature of 29°C. A commercial diet was fed daily to satiation. These fish were used as negative controls (after sacrificed, these asymptomatic fish showed no signs of viral/bacterial infection). The study proposal was reviewed by the grant application committee and granted by the Bureau of Animal and Plant Health Inspection and Quarantine, Council of Agriculture, Taiwan, who specifically approved the experiments.

In order to test the multiplex RT-PCR assay on *ex vivo* specimens, healthy grouper fish were challenged intraperitoneal injection with different pathogens; five-days post-infection RNA was extracted from the challenged fish and subjected to microfluidic chip-based multiplex RT-PCR.

### Egg samples

The grouper eggs were collected from the egg collection net within 1 hour after spawning. Three different broodstock grouper aquaculture farms in southern Taiwan (Fangliao, Shueidiliao, Jiadong; for the location was indicated in Figure S1) were sampled, and the eggs were then transported alive to a laboratory at National Cheng Kung University. The eggs were maintained in a 1×0.5×0.5 m^3^ fish tank with constant aeration and a water temperature of 28±2°C. The experiments were repeated thrice, and the NNV-free eggs were collected as a control group. Samples were collected every three hours until they hatch. The samples were then subjected to real-time PCR for NNV detection.

### Nucleic acid extraction

Each pool of 6 fish (whole fish) was homogenized in liquid nitrogen and subjected to RNA extraction using TRIzol Reagent™ (Molecular Research Center, Inc. Ohio, USA) according to the manufacturer's instructions. RNA samples were used immediately or stored at −80°C.

### cDNA synthesis, PCR and RT-PCR on microfluidic chip

The multiplex RT-PCR assay was performed using microfluidic chips [18]. The microfluidic chip device, which can be easily transported to the fish farms, allowed on-site analysis of the fish samples, and individual PCR signals could be distinguished from the DNA marker (Figure S2). The use of microfluidic chips, including cDNA synthesis, PCR and RT-PCR techniques were described previously [18]. RNA samples were treated with DNase I (New England Biolabs) prior to cDNA synthesis. RNA and cDNA were quantified using an Ultrospec 3300 Pro spectrophotometer (Amersham Biosciences, Piscataway, NJ, USA); nucleic acids were diluted using sheared salmon sperm DNA (5 ng mL^−1^) as a carrier. PCR consisted of denaturation (94°C for 7 min), amplification(40 cycles of 94°C for 40 sec, 60°C for 40 sec and 72°C for 40 sec), and elongation (72°C for 7 min). PCR products were transported automatically to the CE sample reservoir by the last set of micropumps [18].

The primer sets used for amplification were the same as those described previously [18]. IridoMCP-F and IridoMCP-R were designed for iridovirus major capsid protein, VAFA-F and VAFA-R for *V. anguillarum* flagellin A, gMx-F and gMx-R for grouper Mx and NodaRNA1-F and NodaRNA1-R for both nodavirus RdRp (RNA1) and protein B2 (RNA 3, a subgenomic RNA transcribed from the 3′ end of RNA1).

### *In situ* RT-PCR

To confirm our real-time PCR results, the grouper eggs were then subjected to *in situ* RT-PCR. Fifteen-hour-after-spawning-eggs were collected for *in situ* RT-PCR. The eggs were fixed overnight in freshly prepared 10% formaldehyde solution and embedded in paraffin. Four-micrometer-thick sections were cut using a Leica CM 1900 microtome (Leica Microsystems, Nussloch, Germany), mounted on a polylysin-coated slide, deparaffinized in xylene for 5 min, and dehydrated with a graded series of ethanol solutions (30, 50, 70, 85, 95, and 3×100%; each containing 0.85% NaCl). Samples were then stained with hematoxylin-eosin (H&E). *In situ* RT-PCR was modified from a previously described regimen [3], [37] and was done with a DIG probe synthesis kit and DIG nucleic acid detection kit (Roche Applied Science, Mannheim, Germany) according to the manufacturer's instructions. The sections were visualized using an Axiovert 40 microscope (Carl Zeiss, Gottingen, Germany), and images were captured using a SPOT RT3 camera (Spot Imaging Solutions, Sterling Heights, MI).

### *In vivo* infection

Nervous necrosis viruses were isolated from the diseased fish as described previously [3], and *V. anguillarum* was obtained from BCRC (Bioresource Collection and Research Center, Taiwan) 12908. *V. anguillarum* was originally isolated from marine fish. For *in vivo* infection, virus was prepared in 20 µL (1×10^5^ TCID_50_mL^−1^), and challenge was performed by intraperitoneal (i.p.) injection. *V. anguillarum* infection was done by immersion of sea water which contained 10^8^ cells mL^−1^. The bacteria were freshly prepared by growing in Tryptic Soya Broth (TSB)+1.5% NaCl at 28°C for 6 h. Fish were then sampled once the clinical signs were observed and the organs were isolated for detection of virus/bacteria. There were eight experimental groups each containing 6 grouper fish. Group 1 fish were challenged with NNV, group 2 fish were challenged with *V. anguillarum*, group 3 fish were challenged with iridovirus, group 4 fish were challenged with NNV and *V. anguillarum* at the same time, group 5 fish were challenged with NNV and iridovirus at the same time, group 6 fish were challenged with iridovirus and *V. anguillarum* at the same time, group 7 fish were challenged with NNV, iridovirus and *V. anguillarum* at the same time, group 8 fish were used as uninfected controls.

## Supporting Information

Figure S1 **The locations of sampling grouper fish farms in Taiwan.** Taiwan is located between tropical and subtropical regions. The grouper aquacultures are mainly gathered in southern Taiwan due to the grouper fish preferred warm water temperature. Black circles indicate five major regions of sampling grouper aquaculture farms. The open circle indicates the location of National Cheng Kung University (NCKU). (Modified from a map available at http://mapsof.net under a Creative Commons Attribution-ShareAlike 1.0 License.)
(TIF)

Figure S2 **The electropherograms of the RT-PCR products from purified RNA.** The minimum concentration detected on the CE module was 3 copies/µL (Kuo *et al.* unpublished). Each sample includes a mixture of DNA markers and RT-PCR products obtained from the infected grouper. Eleven peaks (corresponding to the DNA markers, added to samples post-amplification) and a single peak of the RT-PCR product from grouper were resolved successfully within 4 min. Panels A–D show the 300-bp, 238-bp, 202-bp, and 171-bp PCR products generated from samples containing iridovirus, *V. anguillarum*, the Mx gene and nodavirus, respectively. **A**. Detection of iridovirus (I) in infected fish sample by microfluidic chip. The primers, derived from the gene encoding the iridovirus major capsid protein, amplify a 300-bp fragment. **B**. Detection of bacteria (V) from infected fish sample by microfluidic chip. The primers, derived from a *V. anguillarum* flagellin A gene sequence, amplify a 238-bp fragment. **C**. Detection of grouper Mx gene (MX) expression in infected fish sample by microfluidic chip. The set of primers amplifies a 202-bp fragment from the grouper Mx. **D**. Detection of nodavirus (NNV) in infected fish sample by microfluidic chip. The primers, derived from the gene encoding RNA-dependent RNA polymerase and protein B2 of nodavirus, amplify a 171-bp fragment.
(TIF)

Figure S3 **Evaluation of multiplex RT-PCR with** ***ex vivo*** **samples (infected fish).** M. DNA marker; lane 1, NNV (nodavirus); lane 2, *V. anguillarum*; lane 3, iridovirus; lane 4, NNV (nodavirus)+*V. anguillarum*; lane 5, NNV (nodavirus)+iridovirus; lane 6, *V. anguillarum*+iridovirus; lane 7, NNV (nodavirus)+*V. anguillarum*+iridovirus.
(TIF)
