# Supplementary figures and images for: Nervous Necrosis Virus Replicates Following the Embryo Development and Dual Infection with Iridovirus at Juvenile Stage in Grouper
Source: PLoS One. 2012 Apr 26;7(4):e36183. doi: 10.1371/journal.pone.0036183 (PMC3338570; doi:10.1371/journal.pone.0036183)

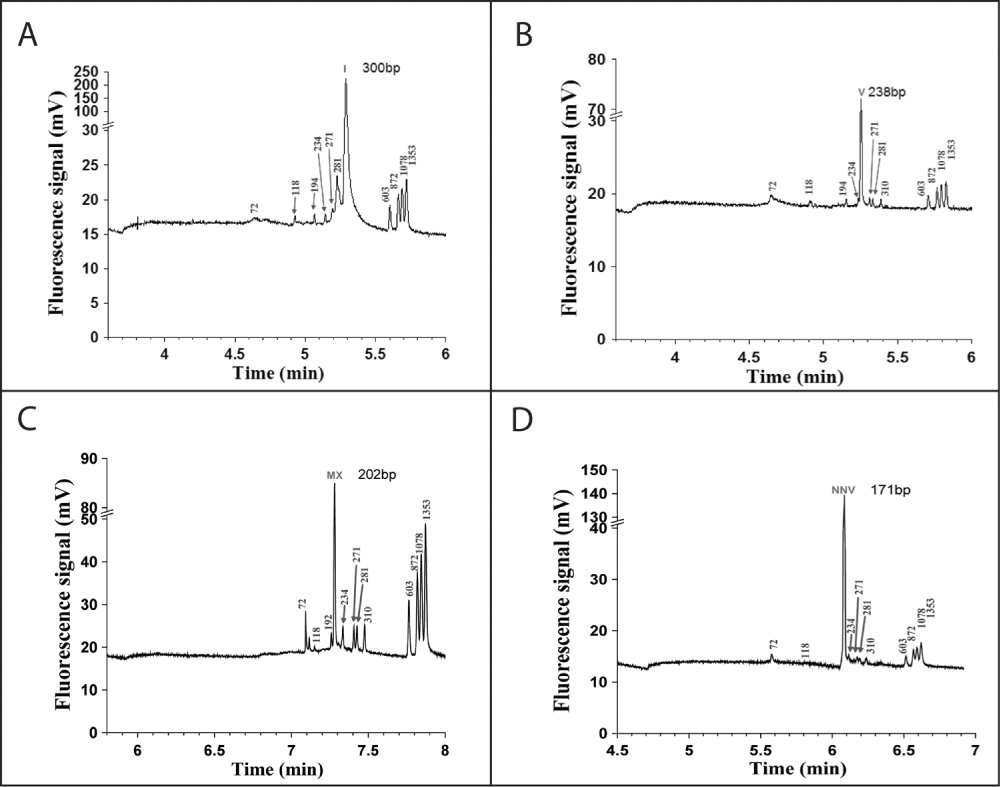

Supplement: Figure S2 — The electropherograms of the RT-PCR products from purified RNA. The minimum concentration detected on the CE module was 3 copies/µL (Kuo et al. unpublished). Each sample includes a mixture of DNA markers and RT-PCR products obtained from the infected grouper. Eleven peaks (corresponding to the DNA markers, added to samples post-amplification) and a single peak of the RT-PCR product from grouper were resolved successfully within 4 min. Panels A–D show the 300-bp, 238-bp, 202-bp, and 171-bp PCR products generated from samples containing iridovirus, V. anguillarum, the Mx gene and nodavirus, respectively. A. Detection of iridovirus (I) in infected fish sample by microfluidic chip. The primers, derived from the gene encoding the iridovirus major capsid protein, amplify a 300-bp fragment. B. Detection of bacteria (V) from infected fish sample by microfluidic chip. The primers, derived from a V. anguillarum flagellin A gene sequence, amplify a 238-bp fragment. C. Detection of grouper Mx gene (MX) expression in infected fish sample by microfluidic chip. The set of primers amplifies a 202-bp fragment from the grouper Mx. D. Detection of nodavirus (NNV) in infected fish sample by microfluidic chip. The primers, derived from the gene encoding RNA-dependent RNA polymerase and protein B2 of nodavirus, amplify a 171-bp fragment. (TIF) [file pone.0036183.s002.tif]

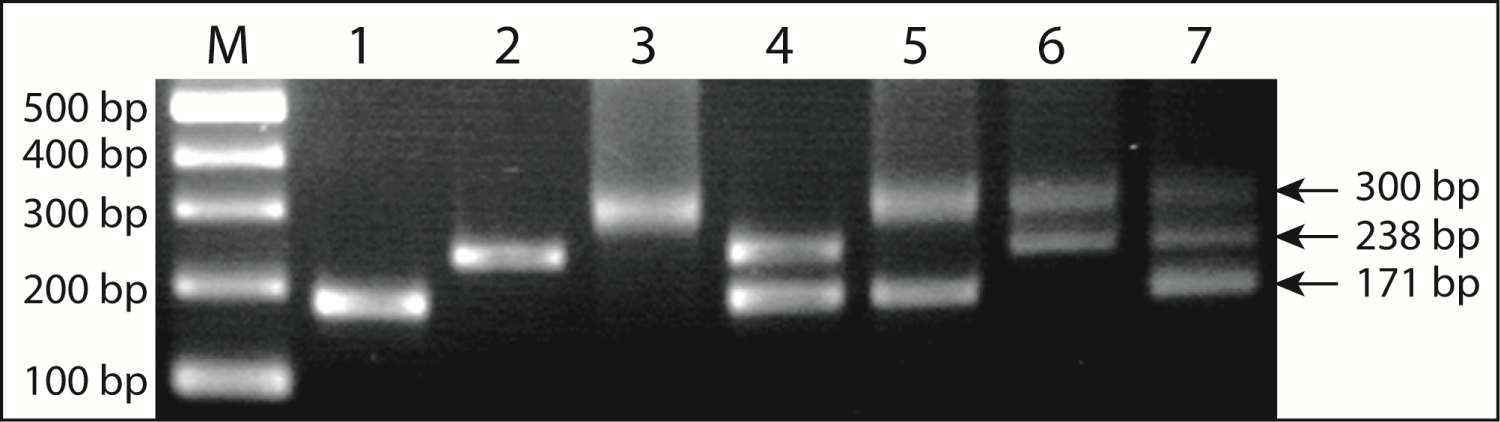

Supplement: Figure S3 — Evaluation of multiplex RT-PCR with ex vivo samples (infected fish). M. DNA marker; lane 1, NNV (nodavirus); lane 2, V. anguillarum; lane 3, iridovirus; lane 4, NNV (nodavirus)+V. anguillarum; lane 5, NNV (nodavirus)+iridovirus; lane 6, V. anguillarum+iridovirus; lane 7, NNV (nodavirus)+V. anguillarum+iridovirus. (TIF) [file pone.0036183.s003.tif]
